# Maternal and neonatal outcomes associated with COVID-19 infection: A systematic review

**DOI:** 10.1371/journal.pone.0234187

**Published:** 2020-06-04

**Authors:** Vinayak Smith, Densearn Seo, Ritesh Warty, Olivia Payne, Mohamed Salih, Ken Lee Chin, Richard Ofori-Asenso, Sathya Krishnan, Fabricio da Silva Costa, Beverley Vollenhoven, Euan Wallace

**Affiliations:** 1 Department of Obstetrics and Gynaecology, Monash University, Clayton, Victoria, Australia; 2 School of Public Health and Preventive Medicine, Monash University, Melbourne, Victoria, Australia; 3 Fetal Monitoring Unit, Monash Medical Centre, Monash Health, Clayton, Victoria, Australia; 4 Melbourne Medical School, The University of Melbourne, Parkville, Victoria, Australia; 5 Department of Pharmacy, University of Copenhagen, København, Denmark; 6 Newborn Services, Western Health, Sunshine, Victoria, Australia; 7 Department of Gynecology and Obstetrics, Ribeirão Preto Medical School, University of São Paulo, Ribeirão Preto, São Paulo, Brazil; Univesity of Iowa, UNITED STATES

## Abstract

**Background:**

COVID-19 has created an extraordinary global health crisis. However, with limited understanding of the effects of COVID-19 during pregnancy, clinicians and patients are forced to make uninformed decisions.

**Objectives:**

To systematically evaluate the literature and report the maternal and neonatal outcomes associated with COVID-19.

**Search strategy:**

PubMed, MEDLINE, and EMBASE were searched from November 1^st^, 2019 and March 28^th^, 2020.

**Selection criteria:**

Primary studies, reported in English, investigating COVID-19-positive pregnant women and reporting their pregnancy and neonatal outcomes.

**Data collection and analysis:**

Data in relation to clinical presentation, investigation were maternal and neonatal outcomes were extracted and analysed using summary statistics. Hypothesis testing was performed to examine differences in time-to-delivery. Study quality was assessed using the ICROMS tool.

**Main results:**

Of 73 identified articles, nine were eligible for inclusion (n = 92). 67.4% (62/92) of women were symptomatic at presentation. RT-PCR was inferior to CT-based diagnosis in 31.7% (26/79) of cases. Maternal mortality rate was 0% and only one patient required intensive care and ventilation. 63.8% (30/47) had preterm births, 61.1% (11/18) fetal distress and 80% (40/50) a Caesarean section. 76.92% (11/13) of neonates required NICU admission and 42.8% (40/50) had a low birth weight. There was one indeterminate case of potential vertical transmission. Mean time-to-delivery was 4.3±3.08 days (n = 12) with no difference in outcomes (p>0.05).

**Conclusions:**

COVID-19-positive pregnant women present with fewer symptoms than the general population and may be RT-PCR negative despite having signs of viral pneumonia. The incidence of preterm births, low birth weight, C-section, NICU admission appear higher than the general population.

## Introduction

On March 11^th^, 2020, the World Health Organisation (WHO) classified the novel coronavirus disease (COVID-19), caused by the SARS-CoV-2 virus, as a global pandemic, highlighting the enormity of the viral outbreak [1].

Typically, most strains of the coronaviruses family are implicated in causing the common cold. Although this paints the picture of a seemingly benign pathogen; the past two decades have witnessed this family of viruses become implicated in two major epidemics: Middle Eastern Respiratory Syndrome (MERS) through MERS–CoV and Severe Acute Respiratory Syndrome (SARS) caused by SARS-CoV [2]. Both viruses share similarities with COVID-19 as they are β-coronaviruses with similar genomic structures. Worryingly, SARS and MERS had case fatality rates (CFR) of 10% and 37% respectively and were responsible for over 10,000 deaths globally. Importantly, they have also been implicated in causing maternal morbidity and mortality [3,4].

At the time of writing, COVID-19 has a global CFR of ~6.4% and has caused more deaths than MERS and SARS combined [1]. Understandably, this raises concerns regarding its effects during pregnancy. This is because pregnancy is associated with physiological changes in women which make them more susceptible to respiratory infections and subsequent rapid progression to respiratory failure. Moreover, the available evidence, based on expert opinion and case series data, suggests expedited delivery to facilitate a 28% reduction in daily oxygen requirements to facilitate maternal respiratory stabilisation during respiratory failure [5–9]. Also, when placentas of SARS-affected women were examined, worsening histopathological features of hypoxic damage were appreciable with increasing time from symptom onset to delivery of the fetus, (termed time-to-delivery (TTD) henceforth) [3,10]. These factors then raise the additional question of whether there were any potential differences in these outcomes with respect to TTD, given the implications it could have for management and prognosis.

Unfortunately, there is a dearth of robust literature to provide guidance to clinicians and patients around these.

Considering this, we performed a systematic review of the literature to better inform clinicians about the maternal and neonatal effects of COVID-19 during pregnancy to facilitate informed decision making.

## Methods

This systematic review was conducted in accordance with the PRISMA guidelines. The study protocol and review were not registered with PROSPERO due to the need for urgent information.

### Search strategy and selection criteria

For the following review, a systematic search was undertaken independently by RW, DS, OP, and MS across PubMed, Ovid Medline and EMBASE from the 1st of November 2019 up until March 28^th^, 2020 with the aid of search information specialist, ROA. Included articles were restricted to those written in the English language.

Search terms utilised across all databases for the study were: (“Covid” OR “coronavirus” OR “SARS-CoV-2” OR “SARS2” OR “2019-nCoV”) AND (“antenatal” OR “prenatal” OR “vertical transmission” OR “pregnancy”). A detailed analysis of the search strategy may be found in S1 Appendix.

The inclusion criteria of studies for the review were articles relating to pregnant women who were positive for COVID-19. All primary designs were considered, including case reports and case series. Given the infancy of the pandemic, urgent need of guidance, and limited higher quality information available on the topic, this was deemed reasonable. Articles from reference lists of studies being screened were also considered suitable for assessment.

Citations were independently screened by RW, OP, and MS for suitability of inclusion and a set of eligible articles was created in accord. Following shortlisting, the full-text articles were read thoroughly by VS, RW, DS, MS, and OP. Inclusion of an article for the review was based on consensus between these authors. Studies that did not relate COVID-19 with antenatal care, vertical transmission, or pregnancy were excluded. Management guidelines/recommendations, opinion pieces, and reviews consisting of case reports/case series were similarly rejected.

### Data analysis

Information was manually extracted from the full-text articles by DS, VS, MS, OP, RW, and SK. If overlapping data was suspected during examination, efforts were made to identify the more comprehensive dataset for inclusion. To ensure complete datasets, authors of the original articles were contacted, however, at the time of submission, no responses were received.

The pregnancy measures of interest were:

- Symptoms on presentation
- Maternal investigations for COVID-19
- Period between initial symptoms presentation and delivery of the baby (TTD)
- Preterm delivery (PTD)
- Mode of delivery
- Fetal distress, as defined by the authors
- Intensive care unit (ICU) admission
- Need for respiratory support
- Maternal mortality rate

The neonatal outcomes of interest were:

- APGAR scores at 1 and 5 minutes
- Birth weight
- Neonatal symptoms at birth which were defined by the authors as the presence of either fever, lethargy, nausea, respiratory symptoms or intolerance to feeding
- Neonatal intensive care unit (NICU) admission
- Neonatal investigations for COVID-19
- Evidence of vertical transmission

### Quality assessment

Quality assessment was independently performed by RW, OP and DS using the Integrated quality Criteria for Review Of Multiple Study designs (ICROMS) tool developed by *Zingg et al*. It is a versatile tool that can evaluate multiple study designs and is widely used in public health [11]. Further details may be found in S2 Appendix.

### Statistical methods

Raw data for the numerical variables in the study were explored for distribution using the Shapiro-Wilk test in tandem with visual plot analysis. Approximately normally distributed data was expressed as mean (±SD) and skewed data was expressed as median (IQR). Categorical variables were expressed as a percentage.

Key baseline characteristics and clinical data were presented as descriptive statistics for the study population where data was available. Meta-analysis or weighted proportions for the outcomes of interest was deemed inappropriate given the sample size and level of missing data.

For the variable TTD, only 12 cases were available for analysis. The outcomes were separated into ≤5 days and >5 days based on the understanding that this was the median time to development of dyspnoea from symptom onset in hospitalised COVID-19 patients [12].

Considering the small sample size, all hypothesis testing to compare proportional differences between the groups were performed using Fisher’s exact test.

For all statistical tests, the assumptions of the test were met, and testing was two-tailed. Furthermore, statistical significance was set at an alpha level of p<0.05.

Statistical analysis was completed using SPSS v25.0.

### Patient and public involvement

Patients and members of the public were not involved in this research at any stage.

## Results

**Fig 1:**
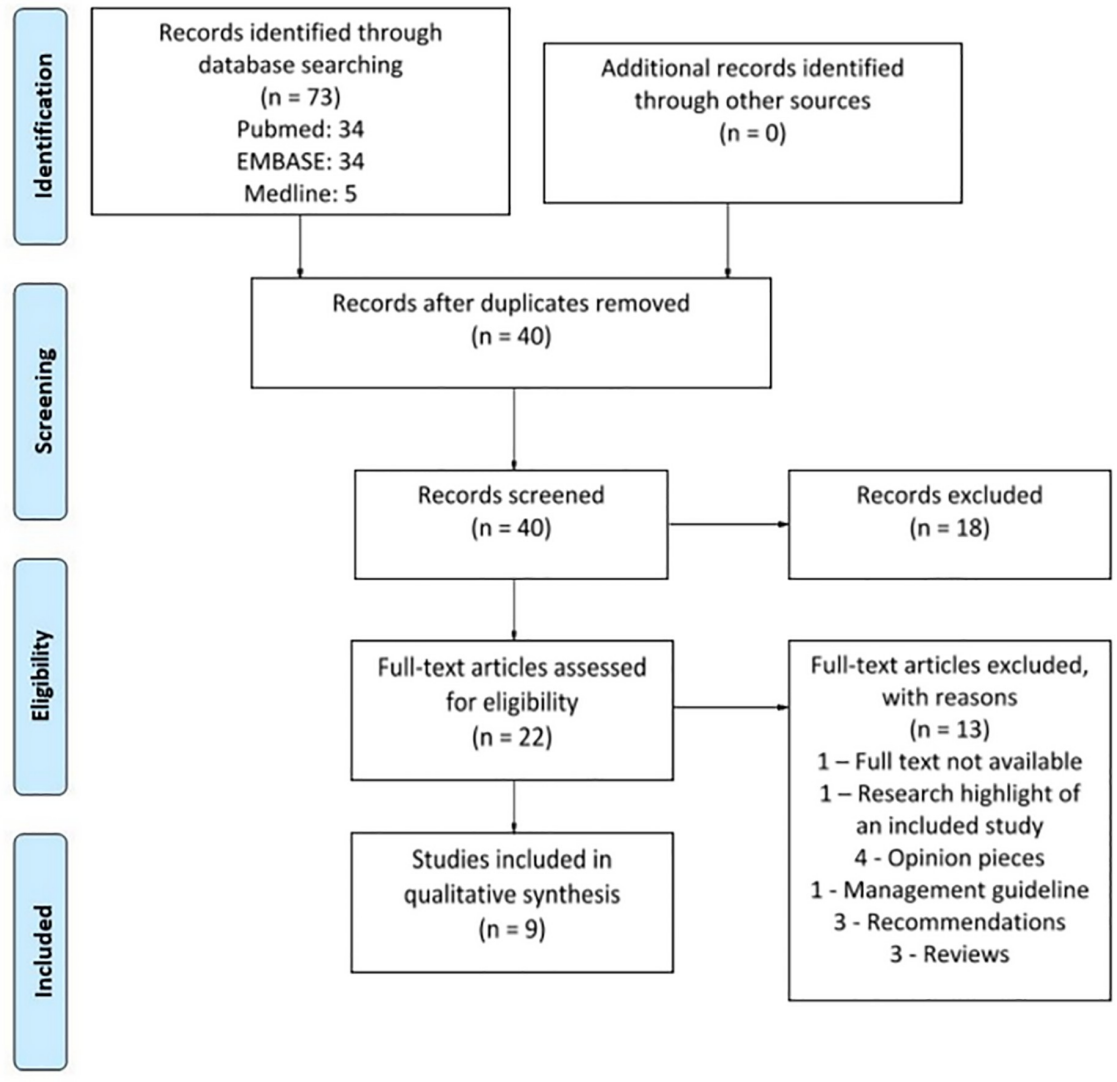
PRISMA flow diagram.

A total of nine studies (n = 92) out of 73 search results were selected for inclusion in this review [13–21]. This is illustrated in Fig 1.

**Fig 2:**
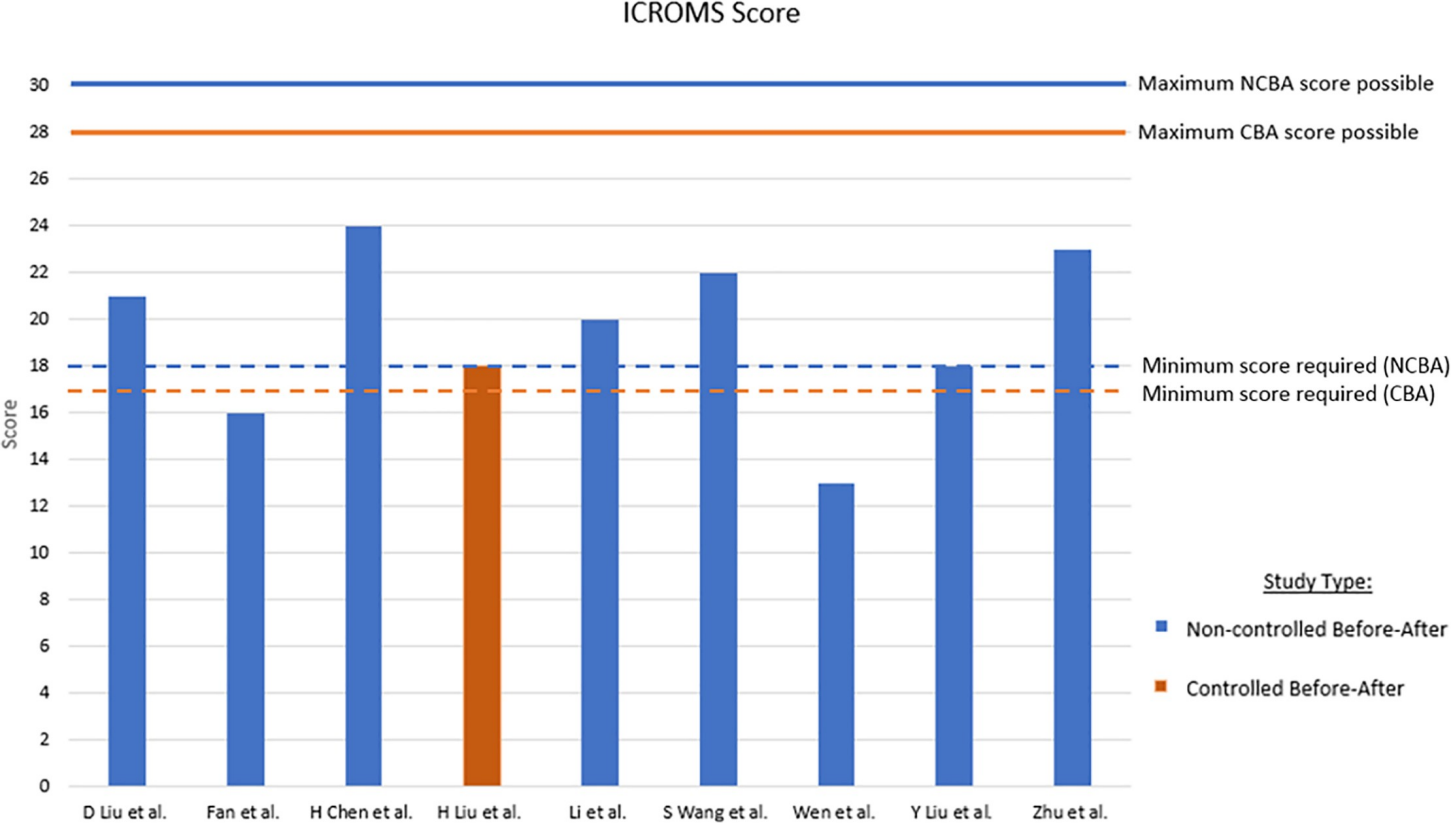
Quality assessment results using ICROMS tool. Minimum required score for NCBA is 18 and CBA is 17. Studies which do not meet this score fail the assessment.

The summary of general characteristics for the included studies are presented in Table 1. The summary statistics for the study are presented in Table 2. Fig 2 provides a summary of the results of the quality assessment process using the ICROMS tool. All studies failed at least one mandatory quality criterion and two, Fan et al. and Wen et al., also failed to meet the minimum required score [14,21].

**Table 1:** General characteristic of included studies.

Author	Study End Date	Study Design	Location	Designated maternal hospital (Y/N/NA)	No. of women	Average age of women (Years)	Average Gestational Age (Weeks)	No. of Neonates
Chen, H. et al. [20]	31st Jan 2020	Retrospective Case Review	Hubei Province, China	Y	9	28	37.3	9
Fan, C. et al. [14]	19th Feb 2020	Case Report	Hubei Province, China	Y	2	31.5	36.5	2
Li, Y. et al. [16]	19th Feb 2020	Case Report	Zhejiang Province, China	Y	1	30	35	1
Liu, D. et al [13]	18th Feb 2020	Case Report	Hubei Province, China	Y	15	32	32	11
Liu, H. et al. [15]	NA	Case Series	Hubei Province, China	Y	41	30	31.4	16
Liu, Y. et al. [19]	25th Feb 2020	Case Series	Guangdong Province, China	Y	13	30	35	10
Wang, S. et al. [17]	NA	Case Report	Hubei Province, China	Y	1	34	40	1
Zhu, H. et al. [18]	5 Feb 2020	Case Series	Hubei Province, China	Y	9	30	NA	10
Wen, R. et al. [21]	20th Feb 2020	Case Report	Qingdao Province, China	NA	1	31	30	0

Y–Yes; N–No; NA–Not available.

**Table 2:** Summary statistical analyses and outcome measures.

Descriptive statistics	(n)/N, summary statistic
Age, yr (mean;SD)	(36) 30.31 ± 3.80
Gestational age at presentation	(27) 35.39 ± 3.51
Stage of pregnancy	(27)
1^st^ trimester	0
2^nd^ trimester	2 (7.41%)
3^rd^ trimester	25 (92.59%)
Symptomatic on presentation	(92) 62 [67.4%]
Fever on admission	(92) 57 [61.96%]
Cough	(92) 35 (38.04%)
Dyspnoea	(83) 10 [12.05%]
Malaise/fatigue	(82) 25 [30.49%]
Myalgia	(28) 6 [21.43]
Sore throat	(50) 6 [12.0%]
Nasal congestion	(4) 2 [50%]
Diarrhoea	(38) 4 [10.43%]
Lymphopenia	(69) 46 [66.67%]
**Maternal Investigations**	
CRP, mg/L (median; SD)	(10) 18.39 (±9.46)
RT-PCR performed	(92) 84 [91.3%]
RT-PCR result positive	(84) 66 [78.6%]
Imaging CT performed	(79) 79 [100%]
Pneumonia found from CT	(79) 78 [98.73%]
**Maternal Outcomes**	
Gestation at delivery, weeks	(13) 37.47 (±1.45)
Pre-term delivery*	(47) 30 [63.83%]
<37 weeks	(13) 6 (46.15%)
<34 weeks	(0) 0 (0%)
Days between symptom and delivery	(12) 4.33 (± 3.08)
ICU admission	(23) 1 (4.35%)
Mode of delivery	(50)
Caesarean section	40 [80.0%]
Vaginal	3 [6.0%]
On-going pregnancy	7 [14.00%]
Symptomatic post-delivery	(72) 28 [38.89%]
Fetal distress (4 ongoing pregnancy)	(18) 11 (61.11%)
Perinatal mortality	(51) [3.92%]
**Neonatal Outcomes**	
Birthweight (mean, SD)	(21) 2743.81 (± 676.34)
Birthweight <2500gm	(21) 9 (42.86%)
APGAR recorded*	(33) 32 (96.97%)
APGAR 1 min*, (median, 25^th^ to 75^th^)	(32) 9 (8–10)
Apgar <7 at 1 min	0
APGAR 5 min*, (median, 25^th^ to 75^th^)	(23) 10 (9–10)
Apgar <7 at 5 min	0
NICU admission *	(13) 11 (76.92%)
Neonate symptomatic of D1*	(23) 12 (52.17%)
Diagnosed with COVID-19*	(21) 1 (4.76%)

NA–Not available.

*ongoing pregnancy excluded, n—number of cases, N—total numbers of pregnancies in analysis

Overall, 67.4% (62/92) of women were symptomatic on presentation. The most common presenting symptoms were fever (61.9%; 57/92), cough (38.4%; 35/92) and malaise/fatigue (30.49%; 25/82). 12.1% of women were dyspneic at presentation (10/83).

In terms of investigations, 91.3% (84/92) of the women had RT-PCR (reverse transcription polymerase chain reaction) to confirm the diagnosis of COVID-19. These were positive in 78.6% (66/84) of cases. Pneumonia was diagnosed in 98.73% of cases (78/79) based on CT findings. Lesions included unilateral/bilateral ground-glass opacities and/or unilateral/bilateral consolidations. 23.1% (17/78) of these women had a negative RT-PCR. Additionally, 66.7% (46/69) of women had lymphopenia.

With respect to maternal outcomes, there were no recorded cases of maternal mortality and 4.3% (1/23) required admission to the ICU. In this case, the patient required mechanical ventilation and extracorporeal membrane oxygenation. Amongst hospitalised patients, 28.13% (9/32) required nasal prongs for supplemental oxygen therapy.

The mean TTD was 4.3±3.08 days (n = 12). 63.83% (30/47) of the sample had a preterm delivery (PTD) as an outcome. Amongst these, 46.15% (6/13) were below 37 weeks and none were below 34 weeks. 80.0% (40/50) of women were delivered by Caesarean Section (C-Section). 61.11% (11/18) of cases reported fetal distress with 4 mothers being undelivered in this setting.

In relation to neonatal outcomes, we uncovered a perinatal mortality rate of 3.92% (2/37) (one neonatal death and one stillbirth) in our sample. The stillbirth occurred to the mother who was admitted to the ICU with no discussion of aetiology. At 1 and 5 minutes, APGAR scores of all neonates were greater than 7. Also, 42.86% (9/21) of delivered babies weighed less than 2500g at time of birth (low birth weight), 76.92% (11/13) required NICU admission for additional care whilst 52.17% (12/23) were symptomatic on day 1 of life.

In our series, 2.7% (1/37) had neonatal COVID-19. It is unclear if this is evidence of vertical transmission or if it was contracted post-delivery due to delayed RT-PCR testing 36 hours from birth. With respect to that, 48.64% (18/37) of neonates had RT-PCR testing. Additional COVID-19 testing included: amniotic fluid (24.32%, 9/37), nasogastric content (12.33%, 6/37), and umbilical cord blood (24.32%, 9/37) which were all negative for COVID-19.

**Fig 3:**
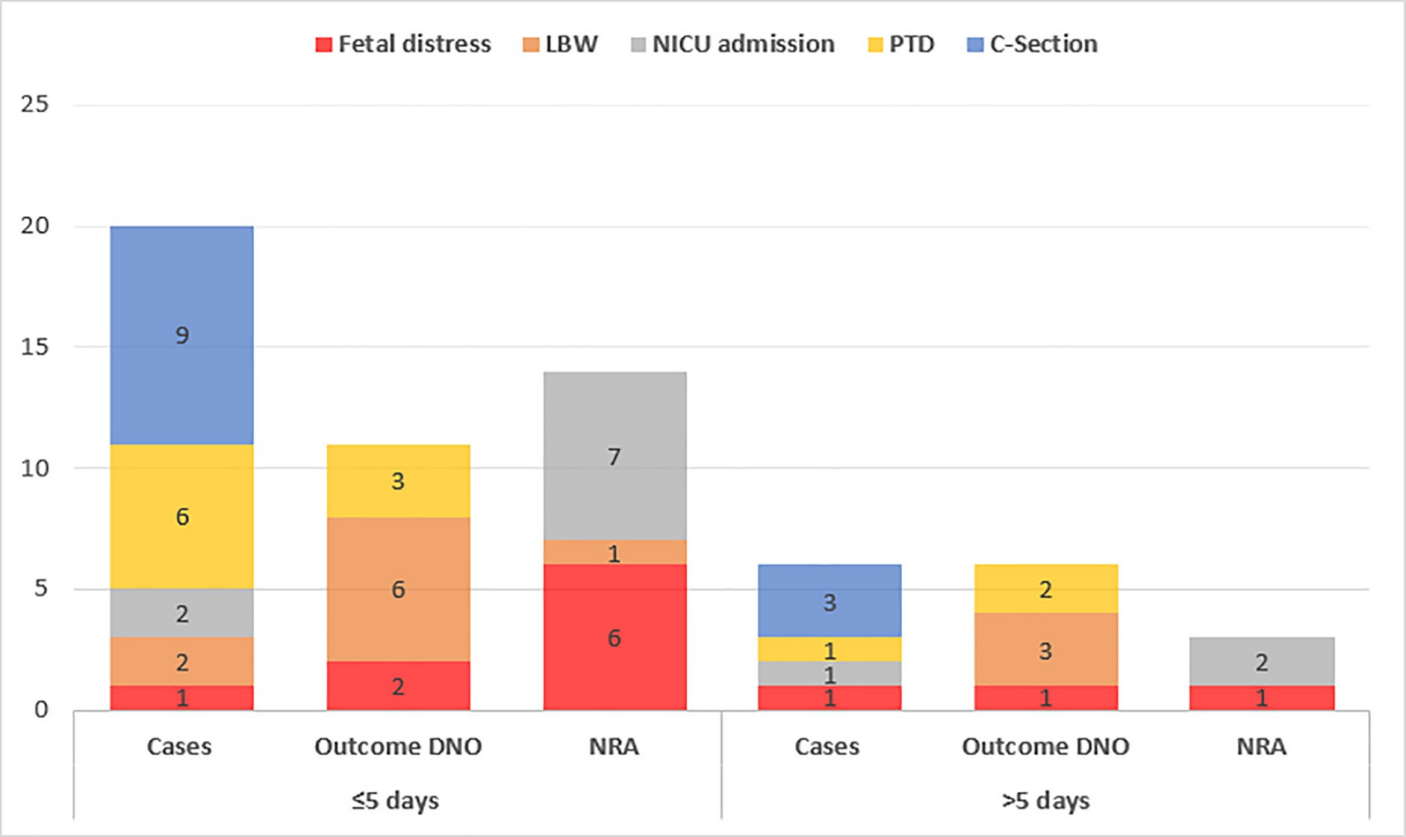
Relationship between maternal and neonatal outcomes, and time to delivery (TTD). DNO—did not occur; NRA—No result available.

Finally, Fig 3 illustrates both maternal and neonatal outcomes with respect to TTD (n = 12). There was no significant difference in the outcomes proportionally when comparing between ≤5 or >5 days (p>0.05).

## Discussion

During pregnancy, women undergo physiological changes that increase their susceptibility to severe respiratory infections and subsequent respiratory failure–a key concern in relation to COVID-19 infections. The mechanisms mediating this are further described in S3 Appendix. Within this discussion, we will also draw comparisons with pregnancy outcomes in analogous *coronaviridae*, SARS and MERS, using two recent rapid reviews [22,23].

### Main findings

Our study sample had a stark difference in their presenting symptoms with a large proportion being asymptomatic and afebrile at presentation. This contrasts with the hospitalised non-pregnant population. A case series (n = 138) in the disease epicentre, Wuhan, illustrated the following symptomatology: fever (98.6%), cough (59.4%), dyspnoea (31.2%), and malaise (69.6%) with ~1% asymptomatic.^12^ One mechanism implicated in this is the disruption of immunological modulation and homeostasis promoted by the placenta by the viral infection, resulting in altered immunological responses and clinical phenotype [24].

66.7% of our sample were lymphopenic. Although associated with a worse prognosis clinically and more severe diseases in the non-pregnant population, it did not appear to translate into poorer outcomes in our study [25]. The incidence of lymphopenia in SARS and MERS were 67% and 50% respectively [22].

Differences in diagnostic reliability of RT-PCR versus CT were identified. In our sample (n = 79), nearly 23.1% were diagnosed based on imaging studies despite negative RT-PCRs. CTs superior sensitivity has already been documented in the general population (98% vs 71%; n = 51; p<0.001) [26]. Although RT-PCR remains the gold standard for diagnosing COVID-19, clinicians should be aware of the potential for false negatives in pregnant women.

With respect to maternal ICU admissions and mortality rates during COVID-19, the available data appears reassuring. In contrast, SARS and MERS respectively had 15–18% and 25–27% mortality rates; 30% and 60% ICU admission rates, and a requirement for mechanical ventilation in 35% and 41% of women [22,23].

The PTB rates in our study appear higher than the baseline in China of 6.7% [27]. Previously it has been reported that 42.7% of PTB in China were iatrogenic in nature but given the lack of indications for delivery, we are unable to discern the level that this is contributory. It begs consideration, that similar PTB rates occur with MERS (13–25.0%), SARS (0–27%) and parturients with severe viral pneumonia as well, thereby implicating viremia in the process [28]. Interestingly, our analysis did not demonstrate any significant differences in PTB rate with respect to TTD [≤5 days vs >5 days (66.67% vs 33.33%; p = 0.52)] from symptoms onset.

Similarly, there was a large proportion of low birth weight (LBW) neonates in our study. Previously, the prevalence of LBW neonates in China were reported as 5.4–6.1% with 61.6–65.3% of PTB babies having LBW too [29,30]. In our sample, 88.9% (8/9) of LBW infants had PTB. In contrast, only one term baby had LBW, which was significantly different (p = 0.02). Although this suggests associations with PTB, it is useful to consider similar trends in women with SARS and severe pneumonia, where there is also an increased likelihood of having a LBW infant [23,31]. One mechanism implicated in this is related to the pre-placental hypoxia which can occur as a result of maternal respiratory compromise with pneumonia/pneumonitis. This can perpetuate a cascade of anti-angiogenic and pro-inflammatory factors promoting endothelial dysfunction, end-organ damage, and placental insufficiency; thereby contributing to relative fetal hypoxemia and eventually hypoxia [32,33]. The high rates of fetal distress in these women enables this argument as it would presumably be mediated similarly.

Our study identified disproportionately high caesarean rates. There are several limitations in interpreting this. Firstly, this may reflect local practice as China has a high baseline caesarean rate (41.5%) [34]. Similarly, one would assume that the high levels of fetal distress may have been contributory. Alternatively, expedited delivery may have been required to facilitate rapid maternal stabilisation and improve ventilation. Importantly, there was no substantial evidence supporting any contraindications towards vaginal delivery. To date, expert consensus has mirrored this finding [35,36].

Our review also demonstrated a high proportion of NICU admissions. These findings were predominantly from the case series by *Zhu et al*. (n = 10) where 60% of the neonates were born premature and all required respiratory support [18]. In this sample, two neonates developed disseminated intravascular coagulopathy and one suffered multi-organ dysfunction which resulted in the subsequent neonatal mortality. Additionally, it was identified that nearly half of these neonates were symptomatic on the first day of life. Although none of the neonatal investigations at birth were positive for COVID-19, *Dong et al*. found that serial RT-PCRs for COVID-19 in neonates may appear negative with the neonate demonstrating delayed serological evidence of infection 3–7 days post-infection [37]. This raises the possibility of a varied clinical phenotype and suggests that early neonatology input and additional serological testing would be beneficial.

This review identified only one case of neonatal COVID-19. However, the evidence of vertical transmission is unclear or if it was contracted post-delivery because the RT-PCR assay was performed when the infant was 36 hours old. Both SARS and MERS have also previously had no cases of vertical transmission recorded [22,23].

### Strengths

The study informs clinicians and patients regarding the risks of COVID-19 during pregnancy using a larger sample size than the case reports available in the literature which lack the ability to draw firm conclusions. Additionally, this study informs and directs attention towards timing of delivery—a key consideration where no comparable literature exists.

### Limitations

The quality of evidence generated is low due to the small sample size, missing data, potential for reporting bias and the quality of included studies. Additionally, generalising the findings is difficult as the whole study population were from China. Also, as this study incorporates data from hospitalised patients alone, it is unclear if similar findings are extrapolatable to the general pregnant population. As the pandemic evolves, an update will be required with improved data sources to address these aspects.

### Interpretation

The interpretation is as follows:

- Maternity caregivers should be aware that COVID-19-positive pregnant women can present with an atypical distribution or no symptoms.
- Hospitalised pregnant women often have pneumonia with COVID-19 infection.
- RT-PCR, although the gold standard, can have false negatives and may not correlate with CT findings of pneumonia. There is however a significant variation in the swab kits being utilised across regions which may limit the generalisability of this data.
- There is no available evidence that expedited delivery changes any maternal or neonatal outcomes. This begs consideration in future work in directing delivery timing in COVID-19-positive women.
- There is no evidence to suggest a contraindication to vaginal delivery.
- The evidence for vertical transmission appears equivocal.
- The findings associated with LBW and fetal distress suggests a role for monitoring fetal growth in mothers affected by COVID-19.
- Early neonatology involvement in a multi-disciplinary approach is prudent given the potential for neonatal/vertical transmission of COVID-19.

## Conclusions

The current evidence suggests that there are low rates of maternal and neonatal mortality as well as ICU admissions associated with COVID-19. COVID-19-positive pregnant women however may present with fewer symptoms than the general population and may have equivocal investigations for the disease. The incidence of PTB, LBW, C-section, NICU admission appear higher than the general population. There may be a role for growth monitoring in these fetuses and a multidisciplinary approach is recommended in their care.

## Supporting information

S1 Appendix Search strategy. (DOCX)

S2 Appendix ICROMS quality assessment tool. (DOCX)

S3 Appendix Physiological analysis of why pregnant women are a vulnerable population. (DOCX)

S4 Appendix PRISMA checklist. (DOC)

## References

[1] WHO, Coronavirus disease SItuation Report 85. 2020, World Health organisation.

[2] Peeri N.C., et al, The SARS, MERS and novel coronavirus (COVID-19) epidemics, the newest and biggest global health threats: what lessons have we learned? International Journal of Epidemiology, 2020. 10.1093/ije/dyaa033 PMC7197734 32086938

[3] Wong S.F., et al, Pregnancy and perinatal outcomes of women with severe acute respiratory syndrome. Am J Obstet Gynecol, 2004 191(1): p. 292–7. 10.1016/j.ajog.2003.11.019 15295381 PMC7137614

[4] Alfaraj S.H., Al-Tawfiq J.A., and Memish Z.A., Middle East Respiratory Syndrome Coronavirus (MERS-CoV) infection during pregnancy: Report of two cases & review of the literature. Journal of Microbiology, Immunology & Infection, 2019 52(3): p. 501–503. 10.1016/j.jmii.2018.04.005 PMC7128238 29907538

[5] Mehta N., et al, Respiratory disease in pregnancy. Best Practice & Research Clinical Obstetrics & Gynaecology, 2015 29(5): p. 598–611. 25997564 10.1016/j.bpobgyn.2015.04.005

[6] GRAVES C.R., Pneumonia in Pregnancy. Clinical Obstetrics and Gynecology, 2010 53(2): p. 329–336. 10.1097/GRF.0b013e3181de8a6f 20436308

[7] Lapinsky S.E., Acute respiratory failure in pregnancy. Obstetric medicine, 2015 8(3): p. 126–132. 10.1177/1753495X15589223 27512467 PMC4935019

[8] Tomlinson M.W., et al, Does delivery improve maternal condition in the respiratory-compromised gravida? Obstet Gynecol, 1998 91(1): p. 108–11. 10.1016/s0029-7844(97)00585-1 9464731

[9] Daily W.H., et al, Beneficial effect of delivery in a patient with adult respiratory distress syndrome. Anesthesiology, 1990 72(2): p. 383–6. 10.1097/00000542-199002000-00027 2301770

[10] Ng W.F., et al, The placentas of patients with severe acute respiratory syndrome: a pathophysiological evaluation. Pathology, 2006 38(3): p. 210–218. 10.1080/00313020600696280 16753741 PMC7131423

[11] Zingg W., et al, Innovative tools for quality assessment: integrated quality criteria for review of multiple study designs (ICROMS). Public Health, 2016 133: p. 19–37. 10.1016/j.puhe.2015.10.012 26704633

[12] Wang D., et al, Clinical Characteristics of 138 Hospitalized Patients With 2019 Novel Coronavirus-Infected Pneumonia in Wuhan, China. Jama, 2020. 10.1001/jama.2020.1585 PMC7042881 32031570

[13] Liu D., et al, Pregnancy and Perinatal Outcomes of Women With Coronavirus Disease (COVID-19) Pneumonia: A Preliminary Analysis. AJR Am J Roentgenol, 2020: p. 1–6. 10.2214/AJR.20.23072 32186894

[14] Fan C., et al, Perinatal Transmission of COVID-19 Associated SARS-CoV-2: Should We Worry? Clinical infectious diseases: an official publication of the Infectious Diseases Society of America., 2020 17. 10.1093/cid/ciaa226 PMC7184438 32182347

[15] Liu H., et al, Clinical and CT imaging features of the COVID-19 pneumonia: Focus on pregnant women and children. J Infect, 2020. 10.1016/j.jinf.2020.03.007 PMC7156118 32171865

[16] Li Y., et al, Lack of Vertical Transmission of Severe Acute Respiratory Syndrome Coronavirus 2, China. Emerging infectious diseases, 2020 26(6). 10.3201/eid2606.200287 PMC7258467 32134381

[17] Wang S., et al, A case report of neonatal COVID-19 infection in China. Clinical infectious diseases: an official publication of the Infectious Diseases Society of America., 2020 12. 10.1093/cid/ciaa225 PMC7108144 32161941

[18] Zhu H., et al, Clinical analysis of 10 neonates born to mothers with 2019-nCoV pneumonia. Transl Pediatr, 2020 9(1): p. 51–60. 10.21037/tp.2020.02.06 32154135 PMC7036645

[19] Liu Y., et al, Clinical manifestations and outcome of SARS-CoV-2 infection during pregnancy. J Infect, 2020. 10.1016/j.jinf.2020.02.028 PMC7133645 32145216

[20] Chen H., et al, Clinical characteristics and intrauterine vertical transmission potential of COVID-19 infection in nine pregnant women: a retrospective review of medical records. The Lancet, 2020 395(10226): p. 809–815. 10.1016/S0140-6736(20)30360-3 PMC7159281 32151335

[21] Wen R., Sun Y., and Xing Q.S., A patient with SARS-CoV-2 infection during pregnancy in Qingdao, China. J Microbiol Immunol Infect, 2020. 10.1016/j.jmii.2020.03.004 PMC7128446 32198004

[22] Dashraath P., et al, Coronavirus Disease 2019 (COVID-19) Pandemic and Pregnancy. American journal of obstetrics and gynecology., 2020 23. 10.1016/j.ajog.2020.03.021 PMC7270569 32217113

[23] Mullins E., et al, Coronavirus in pregnancy and delivery: rapid review. Ultrasound in obstetrics & gynecology: the official journal of the International Society of Ultrasound in Obstetrics and Gynecology., 2020 17. 10.1002/uog.22014 32180292

[24] Silasi M., et al, Viral infections during pregnancy. Am J Reprod Immunol, 2015 73(3): p. 199–213. 10.1111/aji.12355 25582523 PMC4610031

[25] Zhou F., et al, Clinical course and risk factors for mortality of adult inpatients with COVID-19 in Wuhan, China: a retrospective cohort study. Lancet, 2020 395(10229): p. 1054–1062. 10.1016/S0140-6736(20)30566-3 32171076 PMC7270627

[26] Fang Y., et al, Sensitivity of Chest CT for COVID-19: Comparison to RT-PCR. Radiology, 2020: p. 200432 10.1148/radiol.2020200432 32073353 PMC7233365

[27] Chen C., et al, Epidemiology of preterm birth in China in 2015 and 2016: a nationwide survey. The Lancet, 2018 392: p. S73.

[28] Romero R., et al, The role of infection in preterm labour and delivery. Paediatr Perinat Epidemiol, 2001 15 Suppl 2: p. 41–56. 11520399 10.1046/j.1365-3016.2001.00007.x

[29] Tang W., et al, Low birthweight in China: evidence from 441 health facilities between 2012 and 2014. J Matern Fetal Neonatal Med, 2017 30(16): p. 1997–2002. 10.1080/14767058.2016.1236081 27748149

[30] Chen Y., et al, An epidemiological survey on low birth weight infants in China and analysis of outcomes of full-term low birth weight infants. BMC Pregnancy and Childbirth, 2013 13(1): p. 242. 24370213 10.1186/1471-2393-13-242 PMC3877972

[31] Yost N.P., et al, An appraisal of treatment guidelines for antepartum community-acquired pneumonia. Am J Obstet Gynecol, 2000 183(1): p. 131–5. 10.1067/mob.2000.105743 10920320

[32] Hutter D., Kingdom J., and Jaeggi E., Causes and mechanisms of intrauterine hypoxia and its impact on the fetal cardiovascular system: a review. International journal of pediatrics, 2010. 2010: p. 401323–401323. 10.1155/2010/401323 PMC2963133 20981293

[33] Lim W.S., Macfarlane J.T., and Colthorpe C.L., Pneumonia and pregnancy. Thorax, 2001 56(5): p. 398 10.1136/thorax.56.5.398 11312410 PMC1746055

[34] Ming Y., et al, Dissecting the current caesarean section rate in Shanghai, China. Scientific reports, 2019 9(1): p. 2080–2080. 10.1038/s41598-019-38606-7 30765758 PMC6376037

[35] Poon L.C., et al, ISUOG Interim Guidance on 2019 novel coronavirus infection during pregnancy and puerperium: information for healthcare professionals. Ultrasound in Obstetrics & Gynecology. n/a(n/a). 10.1002/uog.22013 PMC7228229 32160345

[36] Chen D., et al, Expert consensus for managing pregnant women and neonates born to mothers with suspected or confirmed novel coronavirus (COVID-19) infection. Int J Gynaecol Obstet, 2020 149(2): p. 130–136. 10.1002/ijgo.13146 32196655 PMC9087756

[37] Dong L., et al, Possible Vertical Transmission of SARS-CoV-2 from an Infected Mother to Her Newborn. JAMA—Journal of the American Medical Association, 2020: p. E1–E3. 10.1001/jama.2020.4621 PMC7099527 32215581

