# Supplementary material for: Maternal and neonatal outcomes associated with COVID-19 infection: A systematic review
Source: PLoS One. 2020 Jun 4;15(6):e0234187. doi: 10.1371/journal.pone.0234187 (PMC7272020; doi:10.1371/journal.pone.0234187)
Supplement: S1 Appendix — (DOCX) [file pone.0234187.s001.docx]

**Appendix S1: Search Strategy**

Focus of paper: Covid in pregnancy

**Search Strategy:**

*MeSH terms:*

Covid-related: Pregnancy related:

- Wuhan coronavirus
- Prenatal
- Antenatal
- Labour (covered by “pregnancy” in mesh tree)
- Pregnancy
- Vertical transmission
- Wuhan seafood market pneumonia virus
- COVID19 virus
- COVID-19 virus
- coronavirus disease 2019 virus
- SARS-CoV-2
- SARS2
- 2019-nCoV
- 2019 novel coronavirus
- 2019 novel coronavirus infection
- COVID19
- coronavirus disease 2019
- coronavirus disease-19
- 2019-nCoV disease
- 2019 novel coronavirus disease
- 2019-nCoV infection

*Implemented search strategy:*

(Covid OR coronavirus OR SARS-CoV-2 OR SARS2 OR 2019-nCoV) AND (antenatal OR prenatal OR vertical transmission OR pregnancy)

*Search conducted til 28/03/2020.*

*Date restriction of 01/11/2019 – 28/03/2020.*

*Excluded:*

- Non-English language studies.
- Studies that do not relate COVID-19 with antenatal care, vertical transmission, or pregnancy.
- Opinion pieces, recommendations and management/care protocols.

**Search Results:**

- Pubmed 34 citations
- Embase 34 citations (post-manual filtration by entry and publication date)
- Ovid Medline 5 results (post-manual filtration by entry and publication date)

**Calculations:**

n(Total) = 34 + 34 + 5 = 73
n(Duplicates) = 33
n(Total – Duplicates) = 73 – 33 = 40
n(Excluded from abstract screen) = 18
n(Included in FT review) = 40 – 18 = 22
n(Excluded from FT Review) = 13
n(Included in paper) = 9
