# Supplementary material for: Maternal and neonatal outcomes associated with COVID-19 infection: A systematic review
Source: PLoS One. 2020 Jun 4;15(6):e0234187. doi: 10.1371/journal.pone.0234187 (PMC7272020; doi:10.1371/journal.pone.0234187)
Supplement: S2 Appendix — (DOCX) [file pone.0234187.s002.docx]

**Appendix S2: ICROMS Quality Assessment Tool**
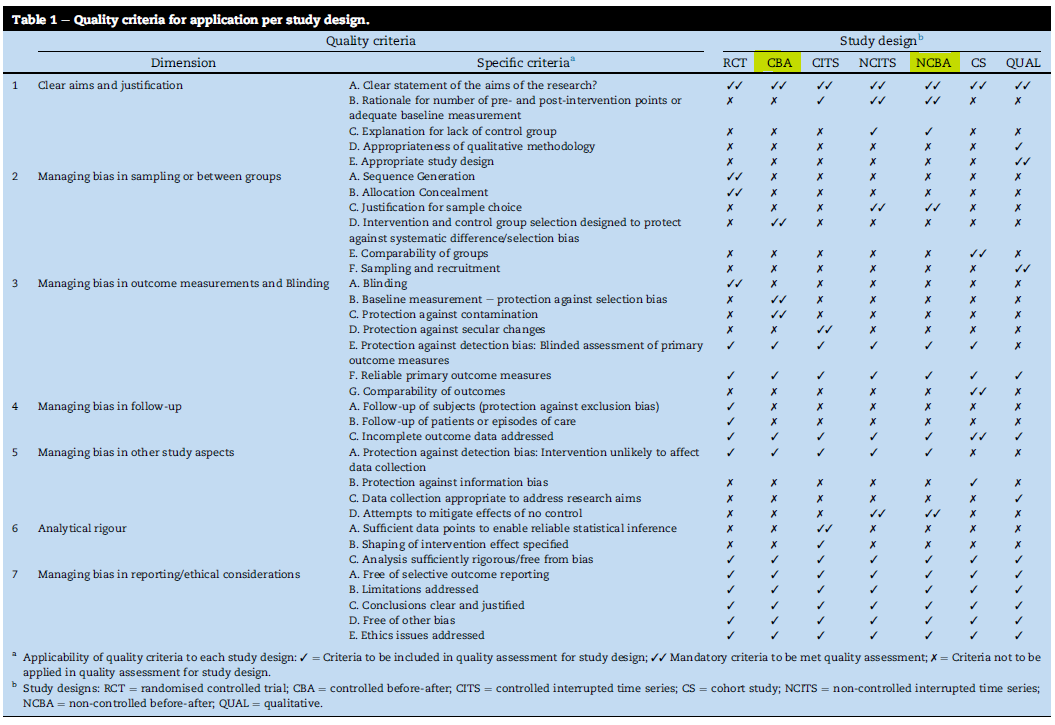


Key notes:

- Using the NCBA section, the maximum available score for a study is 30 points (minimum score required: 18) and for the CBA section, the maximum available points is 28 (minimum score: 17).
- This tool mandates that studies which do not meet the minimum required score or fulfil the mandatory criteria must be excluded from the review. This requirement was excluded due to the circumstances revolving around the COVID-19 crisis and limited available literature.

## NCBA Format:

| **Category** | **Yes (2)/No (0)/Unsure (1)** |
| --- | --- |
| 1. **Clear aims and justification** | |
| a. Clear statement of the aims of the research?* |  |
| b. Rationale for number of pre- and post-intervention points or adequate baseline measurement* |  |
| c. Explanation for lack of control group |  |
| 1. **Managing bias in sampling or between groups** | |
| c. Justification of sample choice* |  |
| 1. **Managing bias in outcome measurements and blinding** | |
| e. Protection against detection bias: Blinded assessment of primary outcome measures |  |
| f. Reliable primary outcome measures |  |
| 1. **Managing bias in follow-up** | |
| c. Incomplete outcome data addressed |  |
| 1. **Managing bias in other study aspects** | |
| a. Protection against detection bias: Intervention unlikely to affect data collection |  |
| d. Attempts to mitigate effects of no control* |  |
| 1. **Analytical rigour** | |
| c. Analysis sufficiently rigorous/free from bias |  |
| 1. **Managing bias in reporting/ethical consideration** | |
| a. Free of selective outcome reporting |  |
| b. Limitations addressed |  |
| c. Conclusions clear and justified |  |
| d. Free of other bias |  |
| e. Ethics issues addressed |  |
| **TOTAL:** |  |

*Mandatory criterion – Fail if not fulfilled.

## CBA Format:

| **Category** | **Yes (2)/No (0)/Unsure (1)** |
| --- | --- |
| 1. **Clear aims and justification** | |
| a. Clear statement of the aims of the research?* |  |
| 1. **Managing bias in sampling or between groups** | |
| d. Intervention and control group selection designed to protect against systematic difference/selection bias* |  |
| 1. **Managing bias in outcome measurements and blinding** | |
| b. Baseline measurement - protection against selection bias* |  |
| c. Protection against contamination* |  |
| e. Protection against detection bias: Blinded assessment of primary outcome measures |  |
| f. Reliable primary outcome measures |  |
| 1. **Managing bias in follow-up** | |
| c. Incomplete outcome data addressed |  |
| 1. **Managing bias in other study aspects** | |
| a. Protection against detection bias: Intervention unlikely to affect data collection |  |
| 1. **Analytical rigour** | |
| c. Analysis sufficiently rigorous/free from bias |  |
| 1. **Managing bias in reporting/ethical consideration** | |
| a. Free of selective outcome reporting |  |
| b. Limitations addressed |  |
| c. Conclusions clear and justified |  |
| d. Free of other bias |  |
| e. Ethics issues addressed |  |
| **TOTAL:** |  |

*Mandatory criterion – Fail if not fulfilled.

# **Results of Quality Assessment using ICROMS**

## Study title/Author: D Liu et al. (NCBA)

| **Category** | **Yes (1)/No (0)/Unsure (U)** |
| --- | --- |
| 1. **Clear aims and justification** | |
| a. Clear statement of the aims of the research? | 2 |
| b. Rationale for number of pre- and post-intervention points or adequate baseline measurement | 2 |
| c. Explanation for lack of control group | 1 |
| 1. **Managing bias in sampling or between groups** | |
| c. Justification of sample choice | 0 |
| 1. **Managing bias in outcome measurements and blinding** | |
| e. Protection against detection bias: Blinded assessment of primary outcome measures | 2 – followed protocols for diagnosis therefore attempted to reduce bias |
| f. Reliable primary outcome measures | 2 |
| 1. **Managing bias in follow-up** | |
| c. Incomplete outcome data addressed | 2 – unlikely to have had an affect |
| 1. **Managing bias in other study aspects** | |
| a. Protection against detection bias: Intervention unlikely to affect data collection | 2 |
| d. Attempts to mitigate effects of no control | 0 |
| 1. **Analytical rigour** | |
| c. Analysis sufficiently rigorous/free from bias | 2 |
| 1. **Managing bias in reporting/ethical consideration** | |
| a. Free of selective outcome reporting | 2 |
| b. Limitations addressed | 0 because the limitation were not in depth enough |
| c. Conclusions clear and justified | 2 |
| d. Free of other bias | 0 |
| e. Ethics issues addressed | 2 |
| **TOTAL:** | 21 |

## Study title/Author: Fan et al. (NCBA)

| **Category** | **Yes (1)/No (0)/Unsure (U)** |
| --- | --- |
| 1. **Clear aims and justification** | |
| a. Clear statement of the aims of the research? | 0 |
| b. Rationale for number of pre- and post-intervention points or adequate baseline measurement | 2 |
| c. Explanation for lack of control group | 0 |
| 1. **Managing bias in sampling or between groups** | |
| c. Justification of sample choice | 0 |
| 1. **Managing bias in outcome measurements and blinding** | |
| e. Protection against detection bias: Blinded assessment of primary outcome measures | 2 |
| f. Reliable primary outcome measures | 2 |
| 1. **Managing bias in follow-up** | |
| c. Incomplete outcome data addressed | 2 |
| 1. **Managing bias in other study aspects** | |
| a. Protection against detection bias: Intervention unlikely to affect data collection | 2 |
| d. Attempts to mitigate effects of no control | 0 |
| 1. **Analytical rigour** | |
| c. Analysis sufficiently rigorous/free from bias | 2 – no real analysis therefore free from bias |
| 1. **Managing bias in reporting/ethical consideration** | |
| a. Free of selective outcome reporting | 2 |
| b. Limitations addressed | 0 |
| c. Conclusions clear and justified | 0 |
| d. Free of other bias | 0 |
| e. Ethics issues addressed | 2 |
| **TOTAL:** | 16 |

## Study title/Author: H Chen et al. (NCBA)

| **Category** | **Yes (1)/No (0)/Unsure (U)** |
| --- | --- |
| 1. **Clear aims and justification** | |
| a. Clear statement of the aims of the research? | 2 |
| b. Rationale for number of pre- and post-intervention points or adequate baseline measurement | 2 |
| c. Explanation for lack of control group | 0 |
| 1. **Managing bias in sampling or between groups** | |
| c. Justification of sample choice | 0 |
| 1. **Managing bias in outcome measurements and blinding** | |
| e. Protection against detection bias: Blinded assessment of primary outcome measures | 2 |
| f. Reliable primary outcome measures | 2 |
| 1. **Managing bias in follow-up** | |
| c. Incomplete outcome data addressed | 2 |
| 1. **Managing bias in other study aspects** | |
| a. Protection against detection bias: Intervention unlikely to affect data collection | 2 |
| d. Attempts to mitigate effects of no control | 2 |
| 1. **Analytical rigour** | |
| c. Analysis sufficiently rigorous/free from bias | 2 – no ‘real’ analysis conducted therefore very unlikely to introduce bias as a result |
| 1. **Managing bias in reporting/ethical consideration** | |
| a. Free of selective outcome reporting | 2 |
| b. Limitations addressed | 2 |
| c. Conclusions clear and justified | 2 |
| d. Free of other bias | 0 – potential of selection bias and the capacity of staff |
| e. Ethics issues addressed | 2 |
| **TOTAL:** | 24 |

## Study title/Author: H Liu et al. (CBA)

| **Category** | **Yes (1)/No (0)/Unsure (U)** |
| --- | --- |
| 1. **Clear aims and justification** | |
| a. Clear statement of the aims of the research? | 2 |
| 1. **Managing bias in sampling or between groups** | |
| d. Intervention and control group selection designed to protect against systematic difference/selection bias | 2 |
| 1. **Managing bias in outcome measurements and blinding** | |
| b. Baseline measurement - protection against selection bias | 2 – by and large the majority of baseline test in different groups were not significantly different |
| c. Protection against contamination | 1 |
| e. Protection against detection bias: Blinded assessment of primary outcome measures | 0 – due to the fact that patients were lost to follow up |
| f. Reliable primary outcome measures | 2 |
| 1. **Managing bias in follow-up** | |
| c. Incomplete outcome data addressed | 1 |
| 1. **Managing bias in other study aspects** | |
| a. Protection against detection bias: Intervention unlikely to affect data collection | 2 |
| 1. **Analytical rigour** | |
| c. Analysis sufficiently rigorous/free from bias | 0 |
| 1. **Managing bias in reporting/ethical consideration** | |
| a. Free of selective outcome reporting | 0 |
| b. Limitations addressed | 2 |
| c. Conclusions clear and justified | 2 |
| d. Free of other bias | 0 |
| e. Ethics issues addressed | 2 |
| **TOTAL:** | 18 |

## Study title/Author: Li et al. (NCBA)

| **Category** | **Yes (1)/No (0)/Unsure (U)** |
| --- | --- |
| 1. **Clear aims and justification** | |
| a. Clear statement of the aims of the research? | 2 |
| b. Rationale for number of pre- and post-intervention points or adequate baseline measurement | 2 |
| c. Explanation for lack of control group | 0 |
| 1. **Managing bias in sampling or between groups** | |
| c. Justification of sample choice | 2 – being a case study they do not need to provide as much of an explanation as to why they include the one patient |
| 1. **Managing bias in outcome measurements and blinding** | |
| e. Protection against detection bias: Blinded assessment of primary outcome measures | 2 |
| f. Reliable primary outcome measures | 2 |
| 1. **Managing bias in follow-up** | |
| c. Incomplete outcome data addressed | 2 |
| 1. **Managing bias in other study aspects** | |
| a. Protection against detection bias: Intervention unlikely to affect data collection | 2 |
| d. Attempts to mitigate effects of no control | 0 |
| 1. **Analytical rigour** | |
| c. Analysis sufficiently rigorous/free from bias | 0 |
| 1. **Managing bias in reporting/ethical consideration** | |
| a. Free of selective outcome reporting | 2 |
| b. Limitations addressed | 2 – although not extensive |
| c. Conclusions clear and justified | 2 – but n=1, therefore is it justified |
| d. Free of other bias | 0 |
| e. Ethics issues addressed | 0 |
| **TOTAL:** | 20 |

## Study title/Author: S Wang et al. (NCBA)

| **Category** | **Yes (1)/No (0)/Unsure (U)** |
| --- | --- |
| 1. **Clear aims and justification** | |
| a. Clear statement of the aims of the research? | 2 |
| b. Rationale for number of pre- and post-intervention points or adequate baseline measurement | 2 |
| c. Explanation for lack of control group | 0 |
| 1. **Managing bias in sampling or between groups** | |
| c. Justification of sample choice | 2 |
| 1. **Managing bias in outcome measurements and blinding** | |
| e. Protection against detection bias: Blinded assessment of primary outcome measures | 2 |
| f. Reliable primary outcome measures | 2 |
| 1. **Managing bias in follow-up** | |
| c. Incomplete outcome data addressed | 2 |
| 1. **Managing bias in other study aspects** | |
| a. Protection against detection bias: Intervention unlikely to affect data collection | 2 |
| d. Attempts to mitigate effects of no control | 0 |
| 1. **Analytical rigour** | |
| c. Analysis sufficiently rigorous/free from bias | 2 |
| 1. **Managing bias in reporting/ethical consideration** | |
| a. Free of selective outcome reporting | 2 |
| b. Limitations addressed | 2 |
| c. Conclusions clear and justified | 2 |
| d. Free of other bias | 0 |
| e. Ethics issues addressed | 0 |
| **TOTAL:** | 22 |

## Study title/Author: Wen et al. (NCBA)

| **Category** | **Yes (1)/No (0)/Unsure (U)** |
| --- | --- |
| 1. **Clear aims and justification** | |
| a. Clear statement of the aims of the research? | 1 |
| b. Rationale for number of pre- and post-intervention points or adequate baseline measurement | 2 |
| c. Explanation for lack of control group | 0 |
| 1. **Managing bias in sampling or between groups** | |
| c. Justification of sample choice | 2 – states that they are presenting the only case of SARS-CoV-2 in pregnancy (for that region of china) |
| 1. **Managing bias in outcome measurements and blinding** | |
| e. Protection against detection bias: Blinded assessment of primary outcome measures | 2 |
| f. Reliable primary outcome measures | 2 |
| 1. **Managing bias in follow-up** | |
| c. Incomplete outcome data addressed | 0 |
| 1. **Managing bias in other study aspects** | |
| a. Protection against detection bias: Intervention unlikely to affect data collection | 2 |
| d. Attempts to mitigate effects of no control | 0 |
| 1. **Analytical rigour** | |
| c. Analysis sufficiently rigorous/free from bias | 0 |
| 1. **Managing bias in reporting/ethical consideration** | |
| a. Free of selective outcome reporting | 2 |
| b. Limitations addressed | 0 |
| c. Conclusions clear and justified | 0 |
| d. Free of other bias | 0 – information bias – as little information was given on testing of the foetus, which would have been important to include |
| e. Ethics issues addressed | 0 |
| **TOTAL:** | 13 |

## Study title/Author: Y Liu et al. (NCBA)

| **Category** | **Yes (1)/No (0)/Unsure (U)** |
| --- | --- |
| 1. **Clear aims and justification** | |
| a. Clear statement of the aims of the research? | 2 – to describe characteristics of covid in pregnancy |
| b. Rationale for number of pre- and post-intervention points or adequate baseline measurement | 2 |
| c. Explanation for lack of control group | 0 |
| 1. **Managing bias in sampling or between groups** | |
| c. Justification of sample choice | 2 |
| 1. **Managing bias in outcome measurements and blinding** | |
| e. Protection against detection bias: Blinded assessment of primary outcome measures | 2 |
| f. Reliable primary outcome measures | 2 |
| 1. **Managing bias in follow-up** | |
| c. Incomplete outcome data addressed | 0 – as three patients did not deliver whilst hospitalised and no attempt was made to follow up on their progress |
| 1. **Managing bias in other study aspects** | |
| a. Protection against detection bias: Intervention unlikely to affect data collection | 2 |
| d. Attempts to mitigate effects of no control | 0 |
| 1. **Analytical rigour** | |
| c. Analysis sufficiently rigorous/free from bias | 0 |
| 1. **Managing bias in reporting/ethical consideration** | |
| a. Free of selective outcome reporting | 2 |
| b. Limitations addressed | 0 |
| c. Conclusions clear and justified | 2 |
| d. Free of other bias | 0 – potential for information bias |
| e. Ethics issues addressed | 2 – not subject to institutional review board as it was an emergency public health outbreak |
| **TOTAL:** | 18 |

## Study title/Author: Zhu et al. (NCBA)

| **Category** | **Yes (1)/No (0)/Unsure (U)** |
| --- | --- |
| 1. **Clear aims and justification** | |
| a. Clear statement of the aims of the research? | 2 |
| b. Rationale for number of pre- and post-intervention points or adequate baseline measurement | 2 |
| c. Explanation for lack of control group | 0 |
| 1. **Managing bias in sampling or between groups** | |
| c. Justification of sample choice | 0 |
| 1. **Managing bias in outcome measurements and blinding** | |
| e. Protection against detection bias: Blinded assessment of primary outcome measures | 2 |
| f. Reliable primary outcome measures | 2 |
| 1. **Managing bias in follow-up** | |
| c. Incomplete outcome data addressed | 2 |
| 1. **Managing bias in other study aspects** | |
| a. Protection against detection bias: Intervention unlikely to affect data collection | 2 |
| d. Attempts to mitigate effects of no control | 0 – attempted to mitigate effect however no real data was given |
| 1. **Analytical rigour** | |
| c. Analysis sufficiently rigorous/free from bias | 2 – rigorous information included |
| 1. **Managing bias in reporting/ethical consideration** | |
| a. Free of selective outcome reporting | 2 |
| b. Limitations addressed | 2 |
| c. Conclusions clear and justified | 2 |
| d. Free of other bias | 1 (unclear) |
| e. Ethics issues addressed | 2 |
| **TOTAL:** | 23 |
