# Supplementary material for: Maternal and neonatal outcomes associated with COVID-19 infection: A systematic review
Source: PLoS One. 2020 Jun 4;15(6):e0234187. doi: 10.1371/journal.pone.0234187 (PMC7272020; doi:10.1371/journal.pone.0234187)
Supplement: S3 Appendix — (DOCX) [file pone.0234187.s003.docx]

**Appendix S3**

**Physiological analysis of why pregnant women are a vulnerable population.**

Pregnancy is a period during which women undergo several physiological changes. These changes increase their **susceptibility to severe forms of respiratory infections and respiratory failure** which is the primary concern in relation to infection with COVID-19.

From a **pulmonary perspective**, women have altered pulmonary volumes across trimesters as a result of diaphragmatic splitting which occurs from the enlarging gravid uterus. As a result, this causes a progressive decline of total lung capacity and ability to clear their respiratory secretions with increasing gestation [1, 2].

Additionally, there are **changes in cellular immunity** which results in the blunting of T-lymphocyte immunity. These include decreased natural killer cell activity, changes in the T-cell population and the proportion circulating T-helper cells, causing a reduced immunological response to prevent maternal recognition of fetal antigens. Expectedly, these changes become more prominent in the second and third trimesters respectively [3, 4].

Finally, the **milieu of hormones** during pregnancy, such as progesterone and human chorionic gonadotropin, remain implicated in facilitating this as well. Apart from inhibiting cell mediated immune function, these trigger changes in gaseous exchange which inherently promotes a state of respiratory alkalosis due to alveolar hypoventilation [1].

**References:**

1. Lim, W.S., J.T. Macfarlane, and C.L. Colthorpe, *Pneumonia and pregnancy.* Thorax, 2001. **56**(5): p. 398-405.

2. LoMauro, A. and A. Aliverti, *Respiratory physiology of pregnancy: Physiology masterclass.* Breathe (Sheffield, England), 2015. **11**(4): p. 297-301.

3. Baley, J.E. and B.Z. Schacter, *Mechanisms of diminished natural killer cell activity in pregnant women and neonates.* The Journal of Immunology, 1985. **134**(5): p. 3042.

4. Sridama, V., et al., *Decreased levels of helper T cells: a possible cause of immunodeficiency in pregnancy.* N Engl J Med, 1982. **307**(6): p. 352-6.
